# Supplementary material for: Breaking the fundamental scattering limit with gain metasurfaces
Source: Nat Commun. 2022 Jul 28;13:4383. doi: 10.1038/s41467-022-32067-9 (PMC9334305; doi:10.1038/s41467-022-32067-9)
Supplement: Supplementary file 1 — Supplementary Information [file 41467_2022_32067_MOESM1_ESM.pdf]

Supplementary Information for

Breaking the fundamental scattering limit with gain metasurfaces

Chao Qian[1,2,3,*], Yi Yang[4], Yifei Hua[1,2,3], Chan Wang[1,2,3], Xiao Lin[1,2,3,*], Tong Cai[1,2,3], Dexin Ye[1], Erping Li[1,2,3], Ido Kaminer[5], and Hongsheng Chen[1,2,3,*]

[1] ZJU-UIUC Institute, Interdisciplinary Center for Quantum Information, State Key Laboratory of Modern Optical Instrumentation, Zhejiang University, Hangzhou 310027, China.

[2] ZJU-Hangzhou Global Science and Technology Innovation Center, Key Lab of Advanced Micro/Nano Electronic Devices & Smart Systems of Zhejiang, Zhejiang University, Hangzhou 310027, China.

[3] Jinhua Institute of Zhejiang University, Zhejiang University, Jinhua 321099, China.

[4] Department of Physics and Research Laboratory of Electronics, Massachusetts Institute of Technology, Cambridge, MA 02139, USA.

[5] Department of Electrical and Computer Engineering, Technion-Israel Institute of Technology, Haifa 32000, Israel.

[*]Corresponding author: chaoq@intl.zju.edu.cn (C. Qian); xiaolinzju@zju.edu.cn (X. Lin); hansomchen@zju.edu.cn (H. Chen)

The PDF file includes:

- Supplementary Note 1: Scattering cross section and scattering width
- Supplementary Note 2: Temporal coupled-mode theory for light scattering
- Supplementary Note 3: Physical mechanism of the microwave gain medium
- Supplementary Note 4: Design of the multi-channel superscatterer
- Supplementary Note 5: Loss/gain influence on multi-channel/single-channel superscatterer
- Supplementary Note 6: Mathematic model of the transient response
- Supplementary Note 7: Details on the transient response of the one-channel superscatterer
- Supplementary Note 8: Transient response of a homogeneous dielectric rod
- Supplementary Note 9: Experimental description

Supplementary Note 1: Scattering cross section and scattering width

As schematically shown in Supplementary Fig. 1, the two-dimensional (2D) cylindrical scatterer considered consists of a dielectric core and an ultrathin metasurface coating surrounded by free space. The ultrathin metasurface can be described by its surface impedance Z_s [S1]. As a conceptual demonstration, we assume that a TE plane wave is incident on the scatterer, propagating along the $+x$ direction, with its electric field occurring along the z direction. In cylindrical coordinates (ρ, ϕ, z), the TE incident plane wave can be represented by an infinite sum of cylindrical waves. According to the Mie scattering theory in cylindrical coordinates, the field components of E_z and H_ϕ in the dielectric and air regions can be expressed as follows [S2]:

$$\begin{cases} E_{z1} = \sum_{m=-\infty}^{\infty} i^m J_m(k_1\rho) e^{im\phi} T_{21,m} \\ H_{\phi 1} = \sum_{m=-\infty}^{\infty} \frac{-k_1}{i\omega\mu_0} i^m \frac{\partial J_m(k_1\rho)}{\partial \rho} e^{im\phi} T_{21,m} \end{cases} \tag{S1}$$

$$\begin{cases} E_{z2} = \sum_{m=-\infty}^{\infty} i^m H_m^{(1)}(k_2\rho) e^{im\phi} R_{21,m} + i^m J_m(k_2\rho) e^{im\phi} \\ H_{\phi 2} = \sum_{m=-\infty}^{\infty} \frac{-k_2}{i\omega\mu_0} \left(i^m \frac{\partial H_m^{(1)}(k_2\rho)}{\partial \rho} e^{im\phi} R_{21,m} + i^m \frac{\partial J_m(k_2\rho)}{\partial \rho} e^{im\phi} \right) \end{cases} \tag{S2}$$

where J_m and $H_m^{(1)}$ are Bessel and Hankel functions, respectively, of the first kind with order m, $k_1 = \frac{\omega}{c}\sqrt{\varepsilon_{r1}}$, $k_2 = \frac{\omega}{c}\sqrt{\varepsilon_{r2}}$, and ε_{r1} and ε_{r2} are the relative permittivity values of region 1 (dielectric) and region 2 (air), respectively. In Eq. (S2), the Hankel component represents the scattering waves, and the Bessel component represents the incident waves. By enforcing the boundary condition at $\rho = \rho_1$:

$$E_{z1} = E_{z2}, \left(H_{\phi 2} - H_{\phi 1}\right) = E_{z2}/Z_s \tag{S3}$$

we obtain:

$$\begin{cases} J_m(k_1\rho) T_{21,m} = H_m^{(1)}(k_2\rho) R_{21,m} + J_m(k_2\rho) \\ \frac{i \cdot k_2}{\omega\mu_2}\left(\frac{\partial H_m^{(1)}(k_2\rho)}{\partial \rho} R_{21,m} + \frac{\partial J_m(k_2\rho)}{\partial \rho} \right) - \frac{i \cdot k_1}{\omega\mu_1} \frac{\partial J_m(k_1\rho)}{\partial \rho} \cdot T_{21,m} = \left(H_m^{(1)}(k_2\rho)\, R_{21} + J_m(k_2\rho) \right)/Z_s \end{cases} \tag{S4}$$

By solving the above two linear equations, the two unknown factors, $R_{21,m}$ and $T_{21,m}$, can be determined. $R_{21,m}$ (denoted as S_m) is the scattering coefficient of the mth angular momentum channel. With the choice of $H_0 = \sqrt{\frac{\text{Watt}}{\text{meter}} \frac{\omega\varepsilon_0}{2}}$, the total scattering cross section, i.e., the total scattered power over the intensity of the incident wave, can be reduced to $C_{\text{sct}} = \sum_{m=-\infty}^{\infty} C_{\text{sct},m}$, with $C_{\text{sct},m} = \frac{2\lambda}{\pi}|S_m|^2$. Typically, in a passive system, $|S_m| \le 1$ (referred to as the single-channel scattering limit), and thus, one always obtains $C_{\text{sct},m} \le \frac{2\lambda}{\pi}$ in the 2D case.

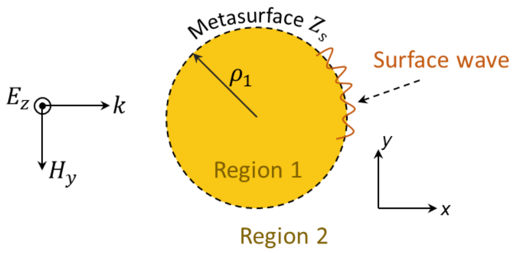

Supplementary Figure 1 | Cross-sectional view of a 2D cylindrical scatterer. A TE plane wave with the electrical field along the z direction impinges on the scatterer encircled by an ultrathin metasurface. The radius of the scatterer is denoted as ρ_1, and the metasurface is described via a surface impedance Z_s.

Apart from the total scattering, more specifically, we also consider the cross section per azimuthal angle as a function of the azimuthal angle ϕ to characterize the far-field radiation pattern [S3]. Given a 2D object, this scattering parameter is also referred to as the scattering width or alternatively as the radar cross section per unit length, which takes the following form:

$$C_{\text{rcs}} = \lim_{\rho\to\infty} 2\pi\rho \frac{|E_z^S|^2}{|E_z^i|^2} = \frac{2\lambda}{\pi} |\sum_{m=0}^{m=\infty} \varepsilon_m S_m \cos(m\phi)|^2 \text{ and } \varepsilon_m = \begin{cases} 1, & m = 0 \\ 2, & m \neq 0 \end{cases} \quad \text{(S5)}$$

The relationship between the scattering cross section and scattering width can be expressed as:

$$C_{\text{sct}} = \frac{1}{2\pi} \int_0^{2\pi} C_{rcs}\, d\phi \quad \text{(S6)}$$

This equation helps us rigorously derive the scattering of a 2D layered cylindrical scatterer and characterize and measure the scattering in the following experiment.

Supplementary Note 2: Temporal coupled-mode theory for light scattering

The temporal coupled-mode theory has successfully modelled a variety of systems, such as optical waveguides and cavities [S4]. The temporal coupled mode can provide an intuitive theoretical framework to model the resonant characteristics of weakly coupled objects. In this section, we provide a general view of resonant scattering based on the temporal coupled-mode theory. As shown in Supplementary Fig. 2, incoming wave h^+ can couple into the resonator or be reflected into outgoing wave h^- (or both). Moreover, energy originating from the resonator must also flow into the outgoing wave h^-. For an obstacle supporting the mth channel, its time-invariant equation relating these quantities in regard to the amplitude A (giving both the magnitude and phase) of the resonance is:

$$\frac{dA}{dt} = (-i\omega_0 - \gamma_{\text{loss}} - \gamma_{\text{leak}})A + \kappa h^+ \quad \text{(S7)}$$

$$h^- = Bh^+ + \eta A \tag{S8}$$

where ω_0 is the resonant frequency, γ_{loss} is the intrinsic loss rate, γ_{leak} is the external leakage rate, B is the background reflection coefficient, and κ and η denote the coupling strength between the resonator and incoming and outgoing waves, respectively. Note that we have suppressed the subscript m in all variables for notation simplicity.

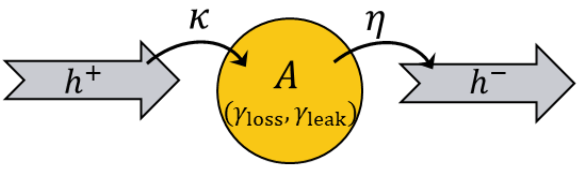

Supplementary Figure 2 | Temporal coupled-mode theory diagram.

With the application of energy conservation and time-reversal symmetry, we can derive the coupling constants κ and η. Considering a simplified case, lossless (i.e., $\gamma_{\text{loss}} = 0$) and no input energy (i.e., $h^+ = 0$), the resonant amplitude decays exponentially, $A(t) = A(0)e^{-i\omega_0 t - \gamma_{\text{leak}} t}$. According to energy conservation, the energy $|A|^2$ leakage rate must be equal to the power of the outgoing wave, i.e.:

$$\frac{d|A|^2}{dt} = -2\gamma_{\text{leak}}|A|^2 = -|h^-|^2 = -\eta\eta^*|A|^2 \tag{S9}$$

which requires that $\eta\eta^* = 2\gamma$. Furthermore, time-reversal transformation can help us solve the other parameters. Assume the resonator is fed with an exponentially growing wave amplitude $A(t) = A(0)e^{-i\omega_0 t + \gamma_{\text{leak}} t}$ without an outgoing wave. Substituting this equation into Eqs. (S7) and (S8), we obtain:

$$\kappa\eta^* = 2\gamma_{\text{leak}} \tag{S10}$$

$$B\eta^* + \eta = 0 \tag{S11}$$

Thus, $\kappa = \eta$. If the system is lossless, the background reflection is $B = e^{i\phi}$ and κ and η are:

$$\kappa = \eta = \sqrt{2\gamma_{\text{leak}}}e^{i(\frac{\phi}{2} + \frac{\pi}{2} - n\pi)} \tag{S12}$$

where n is an arbitrary integer. The reflection coefficient R can be straightforwardly obtained as:

$$R_m = \frac{h^-}{h^+} = e^{i\phi} + \frac{-2\gamma e^{i\phi}}{i(\omega_0 - \omega) + \gamma_{\text{loss}} + \gamma_{\text{leak}}} = \frac{i(\omega_0 - \omega) + \gamma_{\text{loss}} - \gamma_{\text{leak}}}{i(\omega_0 - \omega) + \gamma_{\text{loss}} + \gamma_{\text{leak}}} e^{i\phi} \tag{S13}$$

As expected, in the lossless case (i.e., $\gamma_{\text{loss}} = 0$), the amplitude of R is unity. For a 2D cylindrical scatterer, the scattering coefficient is defined as:

$$S_m = \frac{1}{2}(R - 1) = \frac{1}{2}\frac{(i(\omega_0 - \omega) + \gamma_{\text{loss}})(e^{i\phi} - 1) - \gamma_{\text{leak}}(1 + e^{i\phi})}{i(\omega_0 - \omega) + \gamma_{\text{loss}} + \gamma_{\text{leak}}} \tag{S14}$$

Therefore, we obtain the scattering cross section for an individual channel as:

$$C_{sct,m} = \frac{2\lambda}{\pi}\left|\frac{1}{2}\frac{(i(\omega_0-\omega)+\gamma_{\text{loss}})(e^{i\phi}-1)-\gamma_{\text{leak}}(1+e^{i\phi})}{i(\omega_0-\omega)+\gamma_{\text{loss}}+\gamma_{\text{leak}}}\right|^2 \quad \text{(S15)}$$

In terms of very weak background scattering, $\phi = 0$, the only contribution to the scattering process is that of the resonance. Hence, Eq. (S15) can be written as:

$$C_{sct,m} = \frac{2\lambda}{\pi}\left|\frac{\gamma_{\text{leak}}}{i(\omega_0-\omega)+\gamma_{\text{loss}}+\gamma_{\text{leak}}}\right|^2 \quad \text{(S16)}$$

Supplementary Note 3: Physical mechanism of the microwave gain medium

According to Maxwell's equation, in a causal system, energy conservation can be described by the complex Poynting theorem [S2]:

$$\nabla\cdot(\bar{E}\times\bar{H}^*) = \bar{H}^*\cdot(\nabla\times\bar{E}) - \bar{E}\cdot(\nabla\times\bar{H}^*) \quad \text{(S17)}$$

where $\nabla\times\bar{H}^* = \bar{J}^* + i\omega D^*$ and $\nabla\times\bar{E} = i\omega\bar{B}$. After simplifications, we obtain:

$$\nabla\cdot\bar{S} + i\omega\bar{E}\cdot(\varepsilon(\omega)\bar{E})^* - i\omega\bar{H}^*\cdot(\mu(\omega)\bar{H}) = -\bar{J}^*\cdot\bar{E} \quad \text{(S18)}$$

where $\varepsilon(\omega)$ and $\mu(\omega)$ are the dispersive permittivity and permeability, respectively, and $\nabla\cdot\bar{S} = \nabla\cdot(\bar{E}\times\bar{H}^*)$ is the divergence of the complex Poynting vector, whose real part represents the total power flowing out of an infinitesimally small, closed volume. In a lossy medium, $\text{Re}(\nabla\cdot\bar{S})$ is always negative. $\bar{J}$ is the bulk current density, and it can be expressed as $\bar{J} = \bar{J}_c + \bar{J}_s = \sigma_c(\omega)\bar{E} + \sigma_s(\omega)\bar{E}$, where $\bar{J}_c$ is the conductive current density, $\bar{J}_s$ is the free current density, $\sigma_c(\omega)$ and $\sigma_s(\omega)$ denote the causally dispersive conductivity, and $\bar{J}_s = 0$. Hence, Eq. (S18) can be written as:

$$\nabla\cdot\bar{S} + i\omega\bar{E}\cdot\left(\varepsilon_{eff}(\omega)\bar{E}\right)^* - i\omega\bar{H}^*\cdot(\mu(\omega)\bar{H}) = 0 \quad \text{(S19)}$$

where $\varepsilon_{eff}(\omega) = \varepsilon'_{eff}(\omega) + i\varepsilon''_{eff}(\omega) = \varepsilon(\omega) + i[\sigma_c(\omega)+\sigma_s(\omega)]/\omega$ can be regarded as the effective permittivity of an artificial EM medium. For any passive medium, $\sigma_s(\omega) = 0$ and $\bar{J}_s = 0$. In a general case, as shown in Supplementary Fig. 1, we assume $\bar{E} = \hat{z}E_0e^{ikx}$ and $\bar{H} = \hat{y}E_0e^{ikx}/\eta$, where $k = \omega\sqrt{\varepsilon_{eff}\mu}$ and $\eta = \sqrt{\mu/\varepsilon_{eff}}$ are the complex wavenumber and wave impedance, respectively. These equations are substituted into Eq. (S19) to obtain:

$$\nabla\cdot\bar{S} = \left[-\omega\left(\frac{\varepsilon''_{eff}}{|\varepsilon_{eff}|}+\frac{\mu''}{|\mu|}\right) + i\omega\left(\frac{\varepsilon'_{eff}}{|\varepsilon_{eff}|}-\frac{\mu'}{|\mu|}\right)\right]|\varepsilon_{eff}||\bar{E}|^2 \quad \text{(S20)}$$

$$\text{Re}(\nabla\cdot\bar{S}) = \left[-\omega\left(\frac{\varepsilon''_{eff}}{|\varepsilon_{eff}|}+\frac{\mu''}{|\mu|}\right)\right]|\varepsilon_{eff}||\bar{E}|^2 \quad \text{(S21)}$$

where $\varepsilon''_{eff}(\omega) = [\sigma_c(\omega)+\sigma_s(\omega)]/\omega$, and typically, $\sigma_c(\omega) > 0$. Therefore, for a sufficiently negative σ_s, $\varepsilon''_{eff}(\omega) < 0$, and thus, $\text{Re}(\nabla\cdot\bar{S}) > 0$, i.e., the energy flowing out of an

infinitesimally small, closed volume is higher than the energy flowing into it. In this case, the effective medium described by $\varepsilon_{eff}(\omega)$ can be macroscopically considered a gain medium.

To satisfy the causality law, the physical basis of realizing an artificial gain medium depends on a negative conductance σ_s or negative resistance. In the microwave regime, we can embed negative-resistance components, such as tunnel diodes (TDs) and amplifiers, into metamaterials and metasurfaces [S5–S7]. In conjunction with the optimization algorithm, we can obtain the desired gain medium via artificial metamaterials and metasurfaces.

Supplementary Note 4: Design of the multi-channel superscatterer

A multi-channel superscatterer is a kind of EM device that can greatly boost the total scattering cross section [S8–S14]. By deliberately designing its geometric structure, a superscatterer can engineer an overlap of resonances originating from different plasmonic modes. This device was first theoretically proposed by Fan et al. [S8] and recently experimentally demonstrated by our group in the microwave regime [S9]. Here, as a comparison, we also design a simple multi-channel superscatterer to reveal its transient response. The superscatterer comprises a dielectric cylinder with a relative permittivity of 2.1 coated with an ultrathin metasurface (described by the surface conductivity in a lossy case). The radius of the multi-channel superscatterer is the same as that of the one-channel superscatterer in the main text. Through optimization of the metasurface structure, we realize resonance of the two surface wave modes, as shown in Supplementary Fig. 3. The transient response shown in Fig. 2 in the main text is analysed for this multi-channel superscatterer.

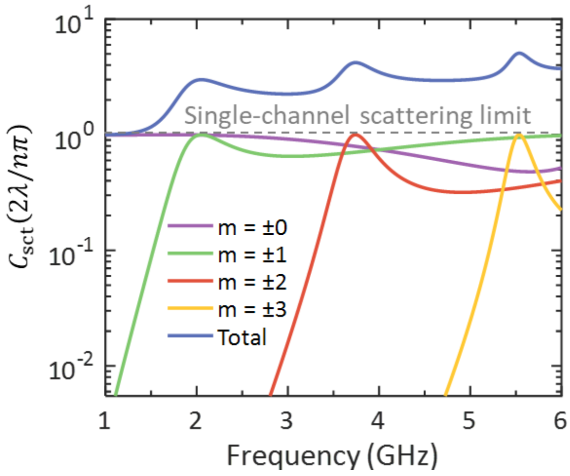

Supplementary Figure 3 | Scattering cross section of the multi-channel superscatterer. The degenerate resonance of confined surface waves emerges simultaneously at 2.2, 3.73, and 5.5 GHz.

Supplementary Note 5: Loss/gain influence on multi-channel/single-channel superscatterer

In the main text, we have clarified that gain is a necessary but insufficient condition for breaking the single-channel scattering limit. Here we take a homogeneous silver rod (with a diameter of $0.223\lambda_p$) under transverse magnetic (TM) waves as a demonstration, where λ_p is the free-space wavelength at plasmon frequency ω_p and $\varepsilon_{im}^{\exp}$ is the imaginary part of the permittivity using experimental data [S15]. As shown in Supplementary Fig. 4a, for conventional multi-channel superscatterer, it is very vulnerable to the material losses. In a gain system (Supplementary Fig. 4b), the total scattering cross section of single-channel superscatterer can be highly boosted by breaking single-channel scattering limit. However, we would like to especially notice that, adding gain does not mean that the single-channel scattering limit (total scattering cross section) is bound to be broken (improved), as exemplified in Supplementary Fig. 4b.

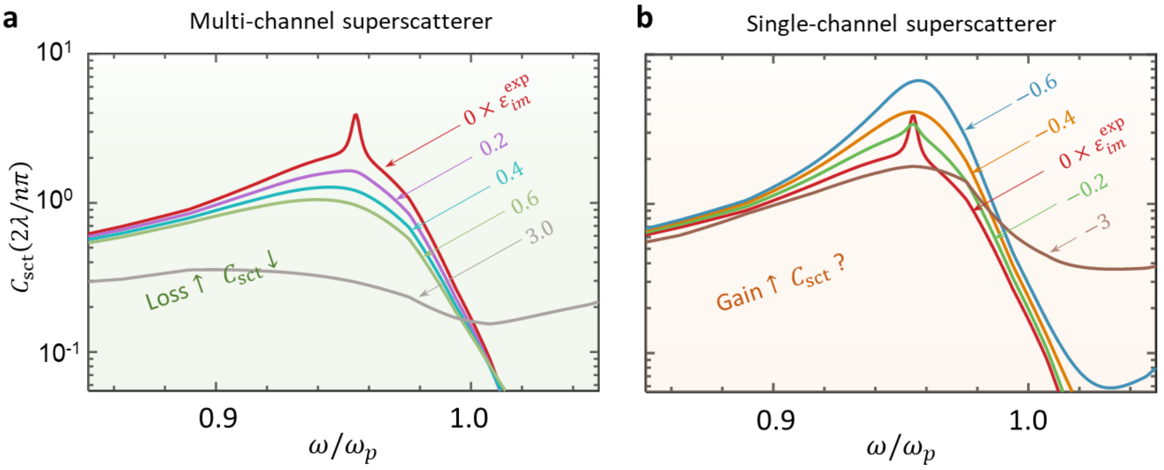

Supplementary Figure 4 | Influence of loss/gain variation on multi-channel/single-channel superscatterer. a and **b**, Scattering cross section related to (**a**) loss and (**b**) gain for conventional multi-channel superscatterer and single-channel superscatterer, respectively.

In addition, based on the single-channel superscatterer (Fig. 3), we vary the gain/loss component to check the scattering cross section (Supplementary Fig. 5). In a lossy system with $Re(Z_s) > 0$, the scattering strength is inversely proportional to the loss. However, in a gain system with $Re(Z_s) < 0$, the scattering strength could increase remarkably. In principle, an infinitely large scattering cross section could be anticipated.

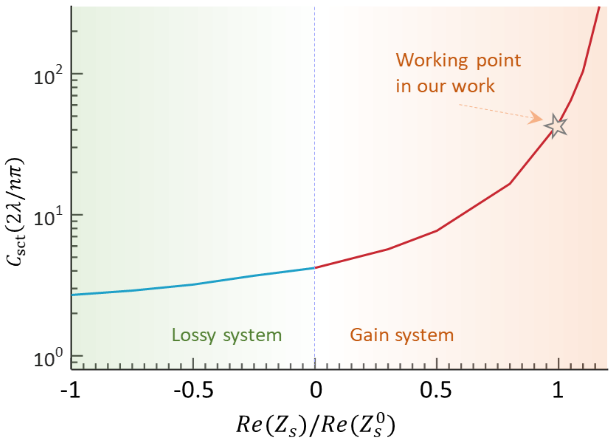

Supplementary Figure 5 |Scattering cross section related to loss/gain. The five-pointed star represents the working point for our single-channel superscatterer. Z_s^0 represents the surface impedance provided by the gain metasurfaces in the single-channel superscatterer. $Re(Z_s^0) = -10.85\ \Omega$ at the working frequency of 3.733 GHz. Here we vary $Re(Z_s)/Re(Z_s^0)$ to change the loss/gain state.

Supplementary Note 6: Mathematic model of the transient response

According to the signal theory [S16], a time-domain signal $g(t)$ (Supplementary Fig. 6a) can be typically expressed into the frequency domain by virtue of a Fourier transform as follows:

$$g(t) = \frac{1}{2\pi}\int_{-\infty}^{\infty} G(\omega)e^{-i\omega t}dt \tag{S22}$$

Based on the above transformation, for another time-domain signal $f(x,y,t) = g(t-(x-l_0)/c)$, its Fourier transform satisfies:

$$f(x,y,t) = \frac{1}{2\pi}\int_{-\infty}^{\infty} G(\omega)e^{i\omega(x-l_0)/c}e^{-i\omega t}dt = \frac{1}{2\pi}\int_{-\infty}^{\infty} G(\omega)e^{ikx}e^{-ikl_0}e^{-i\omega t}dt \tag{S23}$$

where $k = \omega/c$. When $l_0 = -a/2$, it means that $f(x,y,t)$ propagates from the left boundary $x = -a/2$ to the right side (Supplementary Fig. 6b). It should be mentioned that component e^{ikx} exactly corresponds to a monochromatic plane wave. Hence, we can rewrite Eq. (S23) as:

$$f(x,y,t) = \frac{1}{2\pi}\int_{-\infty}^{\infty} G(\omega)E_z^{\mathrm{inc}}(x,y,\omega)e^{-ikl_0}e^{-i\omega t}dt \tag{S24}$$

After the incident wave E_z^{inc} passes through a scatterer, scattered waves are generated denoted as E_z^{sca}. Therefore, the time-domain scattered waves can be expressed as:

$$s(x,y,t) = \frac{1}{2\pi}\int_{-\infty}^{\infty} G(\omega)E_z^{\mathrm{sca}}(x,y,\omega)e^{-ikl_0}e^{-i\omega t}dt \tag{S25}$$

where E_z^{sca} can be rigorously solved based on Mie scattering (Supplementary Note 1).

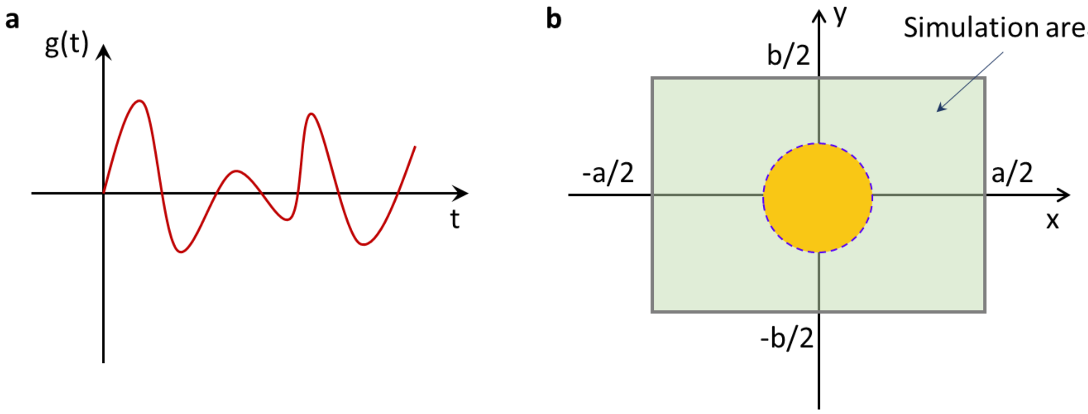

Supplementary Figure 6 | Simulation setup for the transient response. **a**, Random time-domain signal. **b**, Simulation setup, where a time-domain signal impinges on the yellow object from the left boundary $x = -a/2$. The overall simulation area exhibits a dimension of $a \times b$.

As a demonstration, we consider a modulated window signal $e^{-i\omega_0 t + i\frac{\pi}{2}}(u(t) - u(t - 2T_1))$ to impinge the cylindrical scatterer, which satisfies the following Fourier transform:

$$e^{-i\omega_0 t + i\frac{\pi}{2}}(u(t) - u(t - 2T_1)) \rightarrow \frac{2sin(\omega-\omega_0)T_1}{(\omega-\omega_0)} e^{i(\omega-\omega_0)T_1} e^{i\frac{\pi}{2}} \quad \text{(S26)}$$

where $u(t)$ is a step function. In particular, we consider $\omega_0 = 2\pi \cdot 3.733\ \text{GHz}$, $T_0 = \frac{2\pi}{\omega_0} = 0.268\, ns$, and $T_1 = 75T_0 = 20.09\, ns$. Supplementary Fig. 7 shows its Fourier transform and inversely recovered signal. Obviously, the recovered signals are in very high accordance with the original signal, which lays a fundamental basis for the following complicated time-domain calculation.

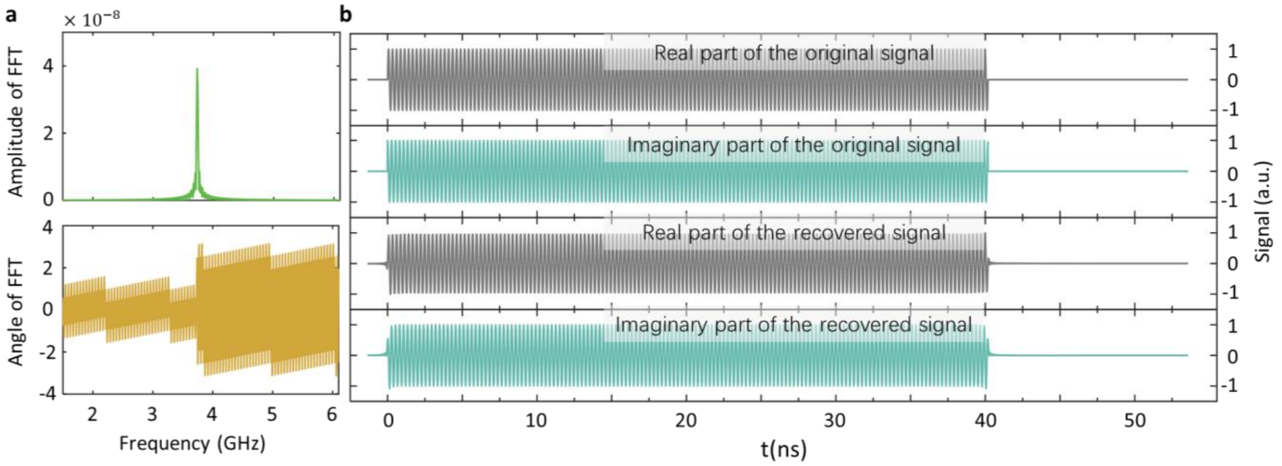

Supplementary Figure 7 | Fourier transform example. **a**, Fourier transform of a modulated window signal. **b**, Original and inversely recovered signals. FFT, fast Fourier transform.

Supplementary Note 7: Details on the transient response of the one-channel superscatterer

We calculated a series of frequency-domain scattered fields (Supplementary Note 1) and converted them into time-domain scattered fields according to Eq. (S25). Figure 2 in the main text is obtained in this manner. To better understand the one-channel superscatterer, we selected more points around the scatterer and plotted their time-varying signals (Supplementary Fig. 8) as a supplement of Fig. 2a. Here, we define that when the received signal at a spatial point remains almost unchanged, its scattered field reaches a stable state [S17]. As shown in Supplementary Fig. 8, at the different points, the time to achieve the steady state varies. Therefore, to precisely judge the energy buildup time of the resonator, we should observe more points and choose the longest time as the final standard.

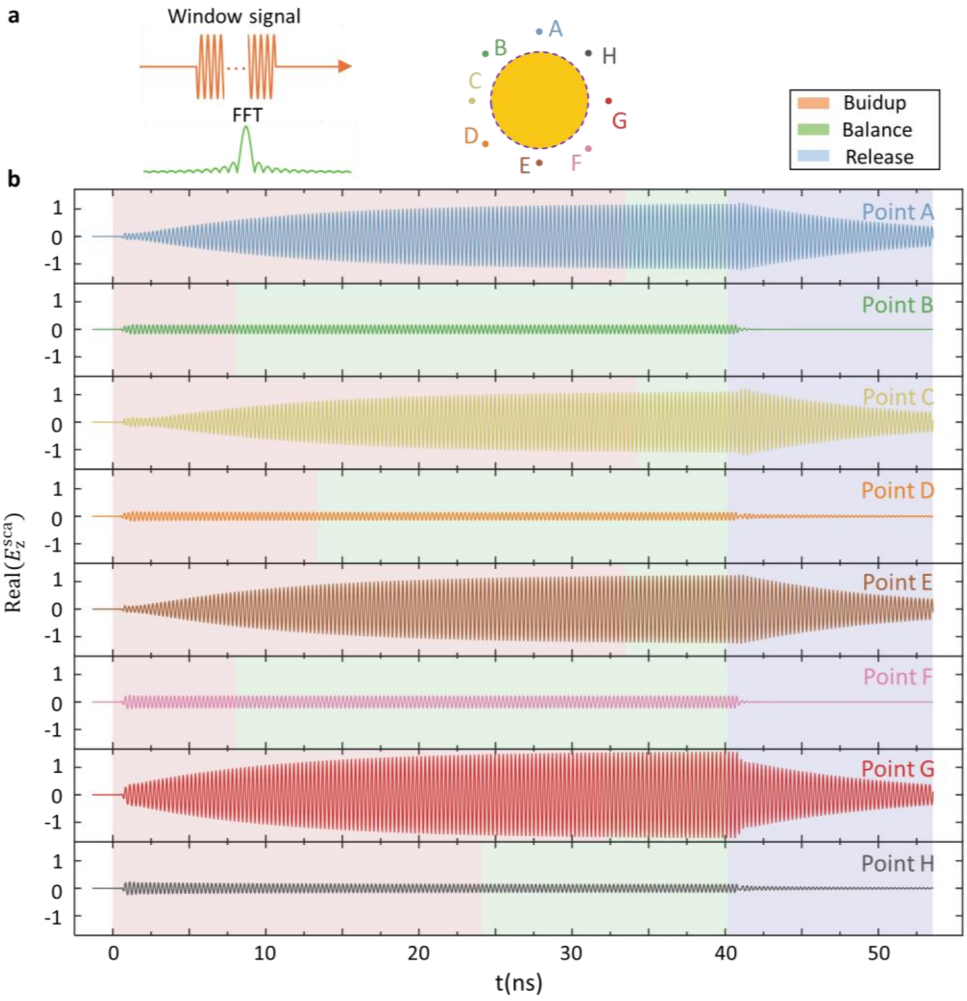

Supplementary Figure 8 | Time-varying signal at eight spatial points around the one-channel superscatterer. a, Schematic of the incident wave (time and frequency domains) and the locations of the eight observation points. **b**, Time-varying signals, one-to-one corresponding to the eight points in **a**. The different points attain different energy buildup and release times.

Supplementary Note 8: Transient response of a homogeneous dielectric rod

In the main text, we analysed the transient response of one-channel and multichannel superscatterers. As a supplement, here, we also analyse a homogeneous dielectric rod with the same radius (14 mm) and relative permittivity (2.1). This dielectric rod achieves a much smaller scattering (Supplementary Fig. 9b), and the time to reach a stable state is extremely short, namely, $\Delta t_3 = 0.8$ ns.

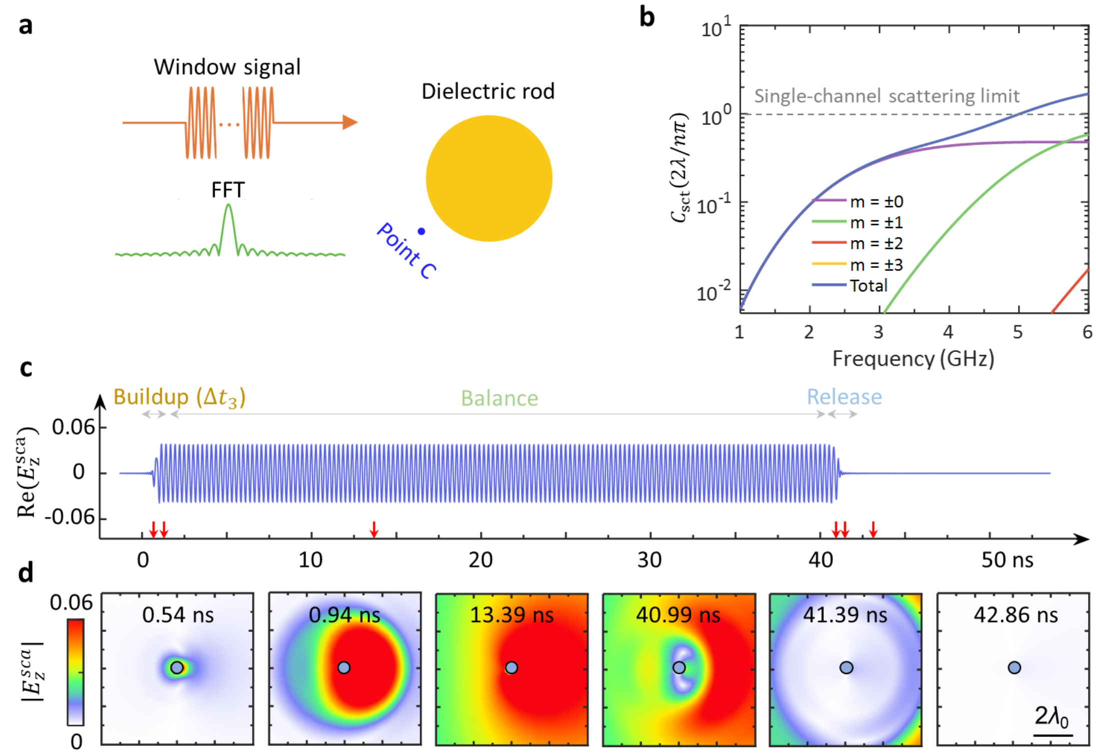

Supplementary Figure 9 | Transient response of a homogeneous dielectric rod. a, A window signal passes through a homogeneous dielectric medium. **b**, Analytical scattering cross section of the dielectric rod. **c**, Time evolution of the electric field at point C. **d**, Time evolution of the spatial electric field.

Supplementary Note 9: Experimental description

Before the experiment, we measured the current-voltage curve of the TDs, as shown in Supplementary Fig. 10. All eight TDs are generally consistent, exhibiting a negative differential resistance from 0.08 V to 0.39 V (manufacturer data). However, a slight shift is observed in the experiment. On this foundation, we welded the TD inside the meta-atom to form a flexible and ultrathin inclusion (with a relative permittivity of 3.4) and coated a subwavelength dielectric cylinder (with a relative permittivity of 2.1), denoted as a multichannel superscatterer.

We experimentally demonstrated the scattering effect of the one-channel superscatterer within a microwave planar waveguide in an anechoic chamber [S18]; see Supplementary Fig. 11. Two metallic plates separated by absorption layers with thicknesses of $h = 18$ mm are adopted to form a parallel plate metallic waveguide. The designed one-channel superscatterer is sandwiched between the upper and lower plates of the waveguide. The length, width and thickness of the upper and lower plates are both 10.00 × 10.00 × 2 mm^3. Before the experiment, we measured the near-field for the pure dielectric rod without gain metasurfaces to check the border effects (Supplementary Fig. 12). A monopole antenna is applied to probe the electric field inside the waveguide (a rectangular window of 272 × 182 mm^2), with a step of 5 mm. The probe antenna and the plate are mounted to a computer-controlled translational stage. The emitting horn antenna is located 2 m away from the waveguide to guarantee a flat wavefront of the incident wave at the entrance of the waveguide. Both the probe antenna and horn antenna are connected to a vector-field network analyser (Ceyear 367cC) for data acquisition.

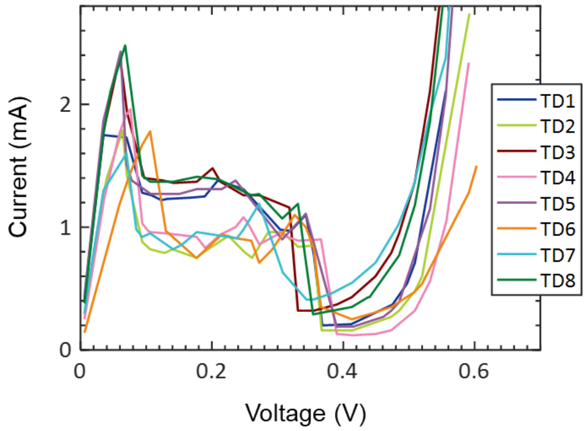

Supplementary Figure 10 | Experimentally measured current-voltage curve of the eight TDs.

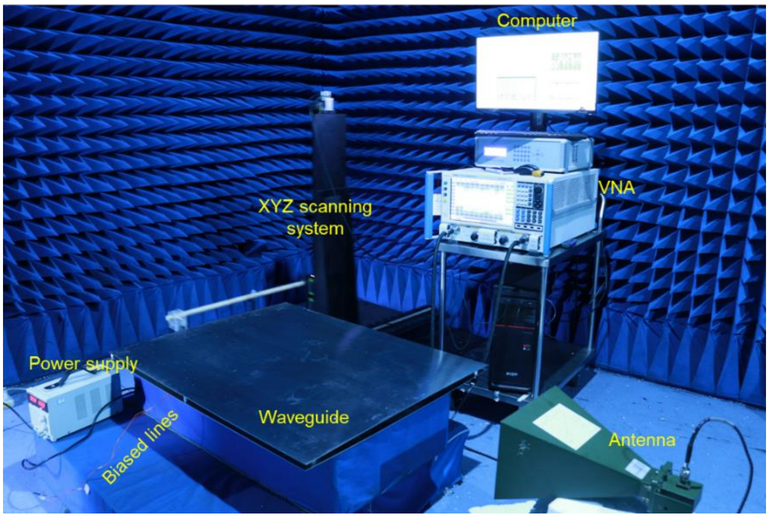

Supplementary Figure 11 | Experimental setup.

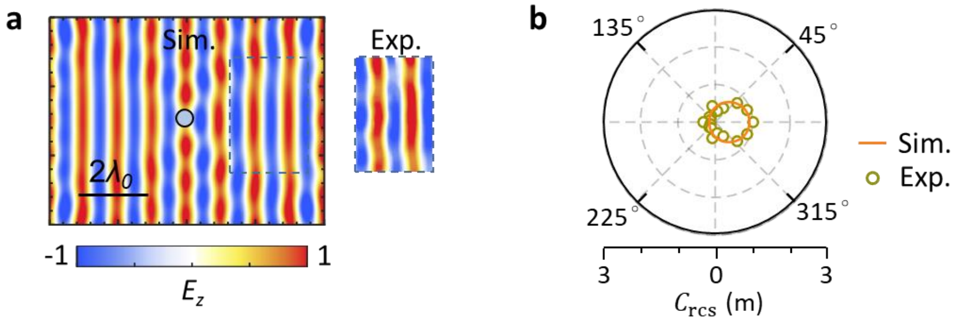

Supplementary Figure 12 | Experimental result of the pure dielectric rod without the gain metasurface. For conceptual demonstration, the measured frequency is 3.55 GHz. **a**, Near-field simulation and measurement of the total field in a region close to the dielectric rod. **b**, Simulated and measured radar cross sections.

Supplementary References

[S1] S. Liu, H. X. Xu, H. C. Zhang, and T. J. Cui, Tunable ultrathin mantle cloak via varactor-diode-loaded metasurface. *Opt. Express* **22**, 13403–13417 (2014).

[S2] W. C. Chew, *Waves and Fields in Inhomogeneous Media* (IEEE, New York, 1995).

[S3] C. A. Balanis, *Advanced Engineering Electromagnetics* (Wiley, New York, 1989).

[S4] R. E. Hamam, A. Karalis, J. D. Joannopoulos, and M. Soljačić, Coupled-mode theory for general free-space resonant scattering of waves. *Phys. Rev. A* **75**, 053801 (2007).

[S5] A. E. Cardin, *et al.* Surface-wave-assisted nonreciprocity in spatio-temporally modulated metasurfaces. *Nat. Commun.* **11**, 1–9 (2020).

[S6] T. Jiang, K. H. Chang, L. Si, L. Ran, and H. Xin, Active microwave negative-index metamaterial transmission line with gain. *Phys. Rev. Lett.* **107**, 205503 (2011).

[S7] T. Yuan, B. I. Popa, and S. A. Cummer, Zero loss magnetic metamaterials using powered active unit cells. *Opt. Express* **17**, 16135–16143 (2009).

[S8] Z. Ruan and S. Fan, Superscattering of light from sub-wavelength nanostructures. *Phys. Rev. Lett.* **105**, 013901 (2010).

[S9] C. Qian, *et al.* Experimental observation of superscattering. *Phys. Rev. Lett.* **122**, 063901 (2019).

[S10] F. Monticone, C. Argyropoulos, and A. Alù, Multilayered plasmonic covers for comblike scattering response and optical tagging. *Phys. Rev. Lett.* **110**, 113901 (2013).

[S11] R. Li, *et al.* Tunable deep-subwavelength superscattering using graphene monolayers. *Opt. Lett.* **40**, 1651 (2015).

[S12] R. Li, X. Lin, S. Lin, X. Liu, and H. Chen, Atomically thin spherical shell-shaped superscatterers based on a Bohr model. *Nanotechnology* **26**, 505201 (2015).

[S13] R. Li, *et al.* Design of ultra-compact graphene-based superscatterers. *IEEE J. Sel. Top. Quantum Electron.* **23**, 4600208 (2017).

[S14] C. Qian, *et al.* Multifrequency superscattering from subwavelength hyperbolic structures. *ACS Photon.* **5**, 1506 (2018).

[S15] E. D. Palik, *Handbook of Optical Constants of Solids* (Academic Press, New York, 1985).

[S16] L. R. Rabiner and B. Gold, *Theory and Application of Digital Signal Processing* (Prentice Hall, 1975).

[S17] C. Qian, *et al.* Transient response of a signal through a dispersive invisibility cloak. *Opt. Lett.* **41**, 4911–4914 (2016).

[S18] H. Chu, *et al.* A hybrid invisibility cloak based on integration of transparent metasurfaces and zero-index materials. *Light Sci. Appl.* **7**, 1–8 (2018).
