# Supplementary material for: Breaking the fundamental scattering limit with gain metasurfaces
Source: Nat Commun. 2022 Jul 28;13:4383. doi: 10.1038/s41467-022-32067-9 (PMC9334305; doi:10.1038/s41467-022-32067-9)
Supplement: Supplementary file 3 — Description of Additional Supplementary Information [file 41467_2022_32067_MOESM3_ESM.pdf]

Description of Additional Supplementary Files

Title: Supplementary Movie 1 (.avi format)

Description: Transient response of the one-channel superscatterer, multi-channel superscatterer, and a homogeneous dielectric rod of the same size. A modulated window incident wave (transverse electric, TE polarized) propagates from left to right. In this process, we provide the time evolution of the scattered field in the entire simulation region and at a specific spatial point.
